# Microbial adherence and surface roughness of denture base materials: An *in vitro* study

**DOI:** 10.6026/973206300211187

**Published:** 2025-05-31

**Authors:** Lalit Kumar, Suranjana Sen, Deepali Agarwal, Megha Gupta, Khadija Anwaar, Shailesh Jain

**Affiliations:** 1 Department of Prosthodontics, CHC, Chandi, Solan, Himachal Pradesh, India; 2 Department of Physiology, DJ College Of Dental Sciences and Research, Modinagar, Uttar Pradesh, India; 3 Department of Microbiology, Institute of Dental Studies and Technology, Kadrabad, Modinagar, Uttar Pradesh, India; 4 Department of Oral and Maxillofacial Surgery, Maharishi Markandeshwar College of Dental Sciences and Research, Maharishi Markandeshwar (Deemed to be University), Mullana, Ambala, Haryana, India; 5 Department of Prosthodontics and Crown & Bridge, Sharda University, Greater Noida, Uttar Pradesh, India; 6 Department of Prosthodontics and Crown & Bridge, Bhojia Dental College, Baddi, Himachal Pradesh, India

**Keywords:** Overdentures, surface roughness, microbial adherence

## Abstract

Implant-supported overdentures have become a widely accepted treatment modality for edentulous patients, offering enhanced retention, stability and comfort compared to conventional complete dentures. Hence, a total of 78 standardized specimens were fabricated and divided into three groups (n = 26 each): Group A (heat-cured PMMA), Group B (CAD/CAM milled resin) and Group C (3D-printed resin). Group B exhibited the lowest surface roughness (0.62 ± 0.12 µm) and microbial adherence (0.41 ± 0.09 OD), followed by Group C (1.25 ± 0.18 µm Ra; 0.68 ± 0.12 OD), while Group A showed the highest values (1.82 ± 0.23 µm Ra; 0.91 ± 0.15 OD). All intergroup comparisons were statistically significant (p < 0.001). A strong positive correlation (r = 0.86, p < 0.001) was observed between surface roughness and microbial adherence. It is concluded that CAD/CAM milled resin offers superior surface smoothness and reduced microbial adherence compared to heat-cured PMMA and 3D-printed resins.

## Background:

Implant-supported overdentures have become a widely accepted treatment modality for edentulous patients, offering enhanced retention, stability and comfort compared to conventional complete dentures [1]. The success rate of implants depends on both proper integration and positioning and suitable properties of denture base material used in prostheses [2]. The clinical success and patient results of treatment highly depend on surface roughness paired with microbial adherence. The key properties influence biofilm development, oral care processes and implant prosthesis longevity. Surface roughness serves as a vital factor which determines how prosthetic materials become colonized by microbes [3]. Guaranteeing microbial longevity begins through surfaces with irregularities because they create protectable cavities which shield microorganisms from mechanical cleaning activities and antimicrobial defense agents. The buildup of these bacterial communities eventually develops into hard-to-remove biofilms that trigger different oral health problems from bad breath to serious conditions affecting implants [4].

The resulting damage to implant stability together with decreased prosthesis durability happens from these conditions. Modern prosthodontics employs denture bases which demonstrate different characteristics in terms of physical makeup and chemical composition and biological performance [5]. Heat-cured polymethyl methacrylate (PMMA) maintains its status as the fundamental choice due to its affordable nature and user-friendliness together with sufficient aesthetic appeal [6]. The smooth surface of standard heat-polymerized acrylics makes them prone to microbial retention because their high roughness and relatively porous nature weakens their resistance to wear over time [7]. Digital dentistry provides manufacturers with two innovative production techniques: CAD/CAM with their precision and reproducibility capabilities and 3D printing for enhanced surface finish results [8]. The manufacturing process of CAD/CAM denture bases produces materials through pre-polymerized resin block milling under controlled environments which results in homogeneous materials with lower residual monomers and smoother surfaces. Studies show that these material characteristics provide two beneficial effects that minimize bacterial attachment during dental exams [9]. Therefore, it is of interest to describe the evaluation of microbial adherence and surface roughness of three different denture base materials in implant supported overdentures.

## Methodology:

The present *in vitro* study aimed to compare the surface roughness and microbial adherence of three different dentures base material sheat-cured PMMA, CAD/CAM milled resin and 3D-printed resin used in implant-supported overdentures. A total of 78 specimens were added in the study fabricated and equally divided into three groups, with each group containing 26 samples.

[1] Group A: 26 specimens fabricated using conventional heat-cured polymethyl methacrylate (PMMA)

[2] Group B: 26 specimens fabricated using CAD/CAM milled denture base resin

[3] Group C: 26 specimens fabricated using 3D-printed denture base resin

## Data collection:

Each specimen was fabricated in a standardized rectangular block shape, measuring approximately 10 mm x 10 mm x 3 mm, to ensure uniformity and allow for accurate surface roughness and microbial testing. For Group A, heat-cured PMMA samples were processed using the compression molding technique. Polymerization was performed in a water bath at 74°C for eight hours, followed by bench cooling. Group B specimens were fabricated by milling pre-polymerized PMMA blocks using a 5-axis CAD/CAM milling machine, ensuring precise and smooth surfaces. Group C specimens were created using a DLP 3D printer with dental-grade photopolymer resin. After printing, samples were post-cured according to the manufacturer's guidelines using a UV polymerization unit. All specimens were finished and polished using a standard protocol with pumice and acrylic polish to mimic the surface condition of clinically delivered prostheses.

## Surface roughness evaluation:

Surface roughness was measured using a contact profilometer. Research groups measured each specimen three times to determine the average surface roughness (Ra) value which was documented in micrometers (µm). Specific laboratory environments controlled all measurements to decrease experimental variations. Surface texture results obtained through Ra values served as quantitative measurements of material surface smoothness which allowed for direct method comparison.

## Microbial adherence testing:

The evaluation of microbial adherence was conducted using Streptococcus mutans (ATCC strain), which is known for its role in initial plaque formation and denture biofilm development. A sterilization process using ethylene oxide gas had to be applied to all specimens before microbial testing took place. Brains heart infusion broth solution with *S. mutans* was used for specimen incubation for twenty-four hours at 37°C under anaerobic environments. The researchers cleaned off non-adherent bacteria from the specimens through gentle washing with sterile phosphate-buffered saline (PBS). The specimens received fixation through 2.5% glutaraldehyde before staining occurred with 0.1% crystal violet solution. The biofilm staining process concluded with 95% ethanol destaining steps which enabled the UV-visible spectrophotometer to determine the optical density at 600 nm wavelength.

## Statistical analysis:

Data were analyzed using SPSS v26. Descriptive statistics including mean and standard deviation were calculated for both surface roughness and microbial adherence values in each group. Intergroup comparisons were performed using one-way analysis of variance (ANOVA). A p-value less than 0.05 were considered statistically significant for all tests conducted.

## Results:

The results showed that Group A (heat-cured PMMA) exhibited the highest surface roughness (1.82 ± 0.23 µm) and microbial adherence (0.91 ± 0.15 OD), indicating a greater propensity for biofilm accumulation. In contrast, Group B (CAD/CAM milled resin) demonstrated the lowest surface roughness (0.62 ± 0.12 µm) and microbial adherence (0.41 ± 0.09 OD), suggesting superior resistance to microbial colonization. Group C (3D-printed resin) presented intermediate values for both parameters (1.25 ± 0.18 µm Ra, 0.68 ± 0.12 OD), reflecting moderate performance (Table 1). The pairwise comparisons using Tukey's post-hoc test revealed statistically significant differences (p < 0.001) in both surface roughness and microbial adherence among all groups. Group A (heat-cured PMMA) had significantly higher surface roughness than both Group B (CAD/CAM milled resin) and Group C (3D-printed resin), with mean differences of 1.20 µm and 0.57 µm, respectively (Table 2). Similarly, microbial adherence was highest in Group A, with notable differences of 0.50 OD and 0.23 OD when compared to Groups B and C. Group A (heat-cured PMMA) showed the widest range and highest median values for both parameters, with a surface roughness median of 1.84 µm and microbial adherence median of 0.93 OD. In contrast, Group B (CAD/CAM milled resin) had the lowest values, with a median Ra of 0.61 µm and microbial adherence of 0.40 OD, indicating a consistently smoother and more biofilm-resistant surface. Group C (3D-printed resin) displayed intermediate values, with a median roughness of 1.23 µm and OD of 0.67, reflecting moderate clinical performance (Table 3). A Pearson correlation analysis demonstrated a strong positive relationship between surface roughness and microbial adherence (r = 0.86, p < 0.001) (Table 4). This indicates that as the surface roughness of denture base materials increases, microbial colonization also tends to rise significantly.

## Discussion:

The present *in vitro* study aimed to compare the surface roughness and microbial adherence of three different dentures base material sheat-cured PMMA, CAD/CAM milled resin and 3D-printed resinused in implant-supported over-dentures. The research indicates substantial variations between the surface characteristics and microbial settlement of the examined materials while the CAD/CAM milled resin material consistently produced the most optimal results [10]. Research findings match previous studies showing that manual finishing of conventional PMMA results in more irregularities and porosities because of polymerization shrinkage [11]. The uneven surfaces create tiny areas for bacteria to remain and form biofilms where early colony groups including Streptococcus mutans effectively proliferate thus serving as key agents in plaque biofilms and peri-implant diseases. The surface roughness measurement of CAD/CAM milled resin (Group B) at 0.62 ± 0.12 µm came in lower than any other treatment methods [12, 13]. The industrial-level manufacturing process for pre-polymerized resin blocks during milling leads to the development of a smooth surface. The processing of these blocks occurs through combination of high temperature and pressure which produces materials with superior surface finish alongside minimal interior voids and enhanced polymer cross-links compared to standard PMMA processing methods [14]. Research shows that CAD/CAM resins exhibit superior biocompatible properties while minimizing microbial attachment which makes them suitable as material choice for implant-supported overdenture applications [15]. The time-efficient fabrication along with customization advantages of 3D printing face resolution constraints of printers alongside characteristic layering which creates surface roughness [16]. The problem with improper polymerization and inconsistent post-processing steps adds to degradation of surface quality [17]. The analysis indicates 3D-printed materials will provide workflow efficiency but practitioners need to conduct extra finishing and curing requirements to optimize product performance. Statistical evaluations proved that these variations were significant. The surface roughness of dental components showed a significant positive relationship (r = 0.86, p < 0.001) with microbial adhesion according to Pearson correlation analysis.

## Conclusion:

The surface characteristics of denture base materials play a significant role in microbial adherence. CAD/CAM milled resin demonstrated the most favourable outcomes, exhibiting the lowest surface roughness and the least microbial colonization. Heat-cured PMMA showed the highest roughness and biofilm retention, while 3D-printed resin displayed intermediate performance. These findings highlight the importance of material selection in prosthodontic practice.

## Figures and Tables

**Table 1:** Mean surface roughness and microbial adherence of denture base materials (n = 26 per group)

**Group**	**Surface Roughness<br>(Ra, µm)**	**Microbial Adherence<br>(OD at 600 nm)**
Group A (Heat-cured PMMA)	1.82± 0.23	0.91± 0.15
Group B (CAD/CAM milled resin)	0.62± 0.12	0.41± 0.09
Group C (3D-printed resin)	1.25± 0.18	0.68± 0.12

**Table 2:** Tukey's Post-Hoc Comparison - Surface Roughness (Ra) and Microbial Adherence (OD 600 nm)

**Surface Roughness (Ra)**	**Mean Difference (µm)**	**p-value**
Group A vs Group B	1.2	< 0.001
Group A vs Group C	0.57	< 0.001
Group B vs Group C	-0.63	< 0.001
Microbial Adherence (OD 600 nm)		
Group A vs Group B	0.5	< 0.001
Group A vs Group C	0.23	< 0.001
Group B vs Group C	-0.27	< 0.001

**Table 3:** Descriptive Statistics for Surface Roughness (Ra) in µm and Microbial Adherence (OD at 600 nm)

**Ra**	**Minimum**	**Maximum**	**Median**
Group A	1.45	2.12	1.84
Group B	0.45	0.79	0.61
Group C	1	1.48	1.23
Microbial Adherence (OD at 600 nm)			
Group A	0.74	1.15	0.93
Group B	0.31	0.59	0.4
Group C	0.55	0.84	0.67

**Table 4:** Pearson correlation between surface roughness and microbial adherence

**Variables Correlated**	**Pearson's r**	**p-value**	**Interpretation**
Surface Roughness vs Microbial Adherence	0.86	< 0.001	Strong Positive Correlation
